# Effects of Fluorescein Staining on Laser In Vivo Confocal Microscopy Images of the Cornea

**DOI:** 10.1155/2012/541974

**Published:** 2012-01-26

**Authors:** Christine W. Sindt, D. Brice Critser, Trudy K. Grout, Jami R. Kern

**Affiliations:** ^1^Department of Ophthalmology and Visual Sciences, University of Iowa Hospitals and Clinics, 200 Hawkins Dr., Iowa City, IA 52242, USA; ^2^Alcon Research Ltd., 6201 South Freeway, Fort Worth, TX 76134, USA

## Abstract

This study was designed to identify whether topical fluorescein, a common ophthalmic tool, affects laser in vivo confocal microscopy of the cornea, a tool with growing applications. Twenty-five eye care specialists were asked to identify presence or absence of fluorescein in 99 confocal micrographs of healthy corneas. Responses were statistically similar to guessing for the epithelium (48% ± 14% of respondents correct per image) and the subbasal nerve plexus (49% ± 11% correct), but results were less clear for the stroma. Dendritic immune cells were quantified in bilateral images from subjects who had been unilaterally stained with fluorescein. Density of dendritic immune cells was statistically similar between the unstained and contralateral stained eyes of 24 contact lens wearers (*P* = .72) and of 10 nonwearers (*P* = .53). Overall, the results indicated that fluorescein staining did not interfere with laser confocal microscopy of corneal epithelium, subbasal nerves, or dendritic immune cells.

## 1. Introduction

In vivo confocal microscopy of the cornea is making the transition from “bench to bedside” [1]. If confocal microscopy will be used in clinical studies or clinical practice, researchers need to know how confocal microscopy might interact with other ophthalmic measurements. 

 Topical sodium fluorescein solution is often used in the evaluation of corneal integrity after contact lens wear [2], in the evaluation of corneal lesions associated with microbial keratitis [3], and in the evaluation of corneal integrity or tear film break-up time in patients with dry eye disease [4]. For ophthalmic sodium fluorescein in a solution with neutral pH, the peak excitation is at 490 nm and the peak emission is at 530 nm [5]. Laser in vivo confocal microscopy uses a wavelength of 670 nm [1]. While these 3 wavelengths appear to be well separated, absorption and emission spectra can have wide bands and secondary peaks. Some researches have investigated the effect of fluorescein on white-light, slit-scanning in vivo confocal microscopy [6, 7], but no studies have yet reported the effect of fluorescein on laser-scanning in vivo confocal microscopy.

 The purpose of this study was to ascertain whether fluorescein staining affects the analysis of laser in vivo confocal microscopy of the central cornea.

## 2. Methods

### 2.1. Image Database

The study population was 48 adults who had provided informed consent for participation in a clinical study that was registered at Clinicaltrials.gov as NCT00804999 and was conducted in accordance with the Declaration of Helsinki. Eligible subjects had been wearing traditional polymer hydrogel or silicone hydrogel contact lenses on a daily wear schedule for at least 2 weeks prior to the qualification examination or were naïve to contact lens wear. Distance visual acuity was required to be correctable with soft contact lenses to 20/30 Snellen or better in each eye in order for a subject to be eligible to enter the study. Exclusion criteria were as follows: one functional eye or a monofit lens; current ocular conditions such as active acute blepharitis, infections, or iritis; any abnormal slit-lamp finding; use of topical ocular medications (prescription or over the counter); or any systemic condition with significant ocular side effects that could adversely affect contact lens wear.

During the study [8], sodium fluorescein (Ful-Glo fluorescein sodium strips; Akorn Inc, Lake Forest, IL) was instilled into at least 1 eye of each subject. By random assignment, some subjects were stained in only 1 eye and some subjects were stained in both eyes (3 : 1 randomization ratio, unilateral : bilateral staining). At approximately 1 to 10 minutes after staining, laser-scanning in vivo confocal microscopy images of the central corneas were captured by a single ophthalmic photographer (D. B. C.) using a Heidelberg Retina Tomograph II with a Rostock Cornea Module (HRT/RCM; Heidelberg Engineering GmbH, Heidelberg, Germany).

### 2.2. Qualitative Analysis

From the overall image database, one investigator selected random representative slides, showing corneas with and without fluorescein, representing the epithelium, the subbasal nerve plexus, and the stroma. Selected images of the epithelium included 19 eyes without fluorescein and 15 eyes with fluorescein. Selected images of the subbasal nerve plexus included 10 eyes without fluorescein and 19 eyes with fluorescein. Selected images of the stroma included 15 eyes without fluorescein and 21 eyes with fluorescein. The images of epithelium, subbasal nerve plexus, and stroma (99 images in total) were presented on slides in a random fashion (i.e., images from similar corneal layers were not grouped together).

During morning rounds at the University of Iowa Hospitals and Clinics, the 99 images were shown as slides to 23 eye care specialists (4 cornea specialists, 6 residents, 4 fellows, 9 faculty members) and 2 medical students. These 25 viewers were asked to identify whether each image showed a cornea with fluorescein or without fluorescein. Data were collected by keypad. Results from the audience were analyzed with a random effects model that accounted for the correlation between a subject's different slides and that was adjusted for anatomical region and presence or absence of sodium fluorescein.

### 2.3. Quantitative Analysis

Dendritic immune cells in the images of the subbasal nerve plexus were tagged in the digital image by a single investigator (C. W. S.) in a masked fashion. The HRT/RCM software then divided the number of the tagged cells by the area of the image (0.16 mm^2^) to yield the density of cells. Cells in each evaluable image were counted twice (by the same investigator) for confirmation of the original count. When available, multiple results from multiple volume scans were averaged per eye before averaging per study group (lens wearers or nonwearers).

From the overall image database of 48 subjects, 34 subjects had been randomized to unilateral fluorescein staining and had bilaterally evaluable confocal scans (not oblique scans). Of those 34 subjects, 24 subjects were habitual wearers of silicone hydrogel or conventional hydrogel contact lenses and 10 subjects were naïve to contact lens wear. Images from these unilaterally stained subjects were analyzed as a subset, and results were compared (with a paired *t*-test) between the fluorescein-stained eyes and the contralateral unstained eyes in each study group (lens wearers or nonwearers). Results are presented as mean ± standard deviation.

## 3. Results

### 3.1. Qualitative Analysis

When the 25 viewers were asked which method they used to identify the presence or absence of fluorescein in the slides, 27% of viewers (7 of 25) said they guessed, 64% of viewers (16 of 25) said they used the luminosity of the slide, and 9% of viewers (2 of 25) said they used some other method. Not all viewers entered responses for every image; number of respondents per image ranged from 21 to 25.

For the overall set of 44 images without fluorescein, viewer responses were not statistically different from expected results with guessing: 51.1% of responses were correct in stating that the images had no fluorescein (95% confidence interval of 47.4% to 54.7% correct). For the overall set of 55 images with fluorescein, viewers were correct in identifying the presence of fluorescein in 45.8% of responses (95% confidence interval of 42.6% to 49.1% correct); this percentage was less accurate than would be predicted by random guessing (45.8% versus expected 50% with guessing, *P* = .01). As shown in Table 1, the misidentification of the presence or absence of fluorescein occurred more frequently with the images of the stroma than with the images of the subbasal nerve plexus or epithelium.

### 3.2. Quantitative Analysis

**Figure 1:**
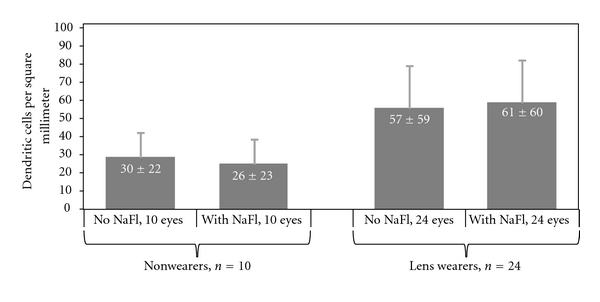
Density of dendritic immune cells identified in the central corneas of fluorescein-stained eyes and of unstained contralateral eyes. Error bars represent 95% confidence interval and are shown unidirectional for clarity. Values are presented as mean ± standard deviation in white text labels. NaFl = sodium fluorescein.

As shown in Figure 1, the presence or absence of fluorescein had no effect on the investigator's ability to identify dendritic immune cells. In subjects who had never worn contact lenses, the mean density of dendritic immune cells was statistically similar (*P* = .53) between the unstained eyes (30 ± 22 cells/mm^2^) and the contralateral stained eyes (26 ± 23 cells/mm^2^). Similarly, in subjects who were habitual wearers of conventional or silicone hydrogel contact lenses, the mean density of dendritic immune cells was statistically similar (*P* = .72) between the unstained eyes (57 ± 59 cells/mm^2^) and the contralateral stained eyes (61 ± 60 cells/mm^2^).

## 4. Discussion

This study demonstrated that topical sodium fluorescein instillation does not impact the qualitative analysis via laser in vivo confocal microscopy of corneal microstructures in the epithelium or the subbasal nerve plexus and does not impact the quantitative analysis via laser in vivo confocal microscopy of dendritic cells in the subbasal nerve plexus layer.

Quantitation via confocal microscopy of central corneal dendritic immune cells has been useful in the analysis of eyes inflamed with vernal keratoconjunctivitis or infectious keratitis [9] and of eyes of healthy contact lens wearers [10]. Similarly, fluorescein staining has been used in the analysis of keratoconjunctivitis sicca [11], of infectious keratitis [3], and of asymptomatic contact lens wearers [2]. Therefore, researchers in these areas may benefit from the knowledge that fluorescein staining, and laser confocal microscopy can be used compatibly.

Examination of the subbasal nerve plexus by confocal microscopy has been useful in the detection of changes in eyes with keratoconus [12] and of eyes recovering from corneal refractive surgery [13]. Similarly, fluorescein staining has been used to evaluate keratoconus [14] and recovery from refractive surgery [15], including dry eye after refractive surgery [16, 17]. The results of this study indicate that researchers in these fields can use fluorescein instillation and laser confocal microscopy as complementary tools in their ophthalmic assessments.

In vivo confocal microscopy images have revealed hyperreflective cells in the epithelium that increase in number in association with certain combinations of contact lenses and lens care solution (silicone hydrogel lenses and a solution that contained polyhexamethylene biguanide), significantly more than in association with other lens/solution combinations (e.g., silicone hydrogel lenses and a solution that contained polyquaternium-1 and myristamidopropyl dimethylamine) [7]. Different levels of cytoplasmic reflectivity from one epithelial cell to another within a confocal micrograph are thought to represent various stages of progression toward cell death [18]. Similarly, animal studies have indicated that punctate fluorescein staining of the corneal epithelium corresponded to the presence of damaged epithelial cells [19], and clinical studies have revealed that wearers of silicone hydrogel lenses that were conditioned with a solution containing polyhexamethylene biguanide had excessive corneal fluorescein staining, while wearers of silicone hydrogel lenses conditioned with comparator solutions had minimal corneal fluorescein staining [20–22]. For these solution-induced corneal changes, other researchers have already demonstrated that no significant association existed between the number of hyperreflective cells revealed by white-light, slit-scanning confocal microscopy and the presence or absence of sodium fluorescein [7]; our research supports theirs in finding that fluorescein staining and laser-scanning confocal microcopy can yield complementary and compatible results.

In contrast to the results in the current study for the epithelium, for the subbasal nerve plexus, and for dendritic cells, the results were less clear in establishing independence between fluorescein staining and confocal microscopy for the corneal stroma. Further investigation is merited for this layer.

The current study was limited to small numbers of samples and to healthy eyes. Moreover, the amount of fluorescein delivered by the strips could vary, and the duration between staining and microscopy was not tightly standardized; instilling a more uniform amount of fluorescein solution and capturing the images after a more consistent period of time could have controlled the experiment better. Still, we feel that the results support the conclusion that researchers can use laser in vivo confocal microscopy and fluorescein staining as complementary tools that do not interfere with each other for the analysis of the epithelium, the subbasal nerve plexus, and dendritic immune cells.

## Figures and Tables

**Table 1:** Percentage of respondents who were correct or incorrect in identifying the presence or absence of fluorescein in confocal microscopy images of the cornea. Results are mean ± standard deviation for results from 21 to 25 respondents per image (up to 4 respondents abstained from responding to various images).

Sample	Percentage of respondents correct per image, %	Percentage of respondents incorrect per image, %
Epithelium images		
No fluorescein, *n* = 19 images	44 ± 17	56 ± 17
With fluorescein, *n* = 15 images	53 ± 12	47 ± 12
*Overall, epithelium, n* = 34* images *	*48 ± 14 *	*52 ± 15 *
Subbasal nerve plexus images		
No fluorescein, *n* = 10 images	48 ± 14	52 ± 14
With fluorescein, *n* = 19 images	50 ± 10	50 ± 10
*Overall, nerves, n* = 29* images *	*49 ± 11*	*51 ± 11*
Stroma images		
No fluorescein, *n* = 15 images	60 ± 18	40 ± 18
With fluorescein, *n* = 21 images	36 ± 20	64 ± 20
*Overall, stroma, n* = 36* images *	*46 ± 23*	*54 ± 23 *

All 3 corneal layers, *n* = 99 images	48 ± 17	52 ± 17
